# Silver Nanoparticles-Chitosan Nanocomposites: A Comparative Study Regarding Different Chemical Syntheses Procedures and Their Antibacterial Effect

**DOI:** 10.3390/ma17051113

**Published:** 2024-02-28

**Authors:** Dan Chicea, Alexandra Nicolae-Maranciuc, Liana-Maria Chicea

**Affiliations:** 1 Research Center for Complex Physical Systems, Faculty of Sciences, Lucian Blaga University of Sibiu, 550012 Sibiu, Romania; 2 Institute for Interdisciplinary Studies and Research (ISCI), Lucian Blaga University of Sibiu, 550024 Sibiu, Romania; 3 Faculty of Medicine, Lucian Blaga University of Sibiu, 550169 Sibiu, Romania; liana.chicea@ulbsibiu.ro

**Keywords:** silver nanoparticles, chitosan, nanocomposite, chemical synthesis, particle sizing, DLS, AFM, antibacterial effect, UV–VIS, FT-IR

## Abstract

Nanocomposites based on silver nanoparticles and chitosan present important advantages for medical applications, showing over time their role in antibacterial evaluation. This work presents the comparative study of two chemical synthesis procedures of nanocomposites, based on trisodium citrate dihydrate and sodium hydroxide, using various chitosan concentrations for a complex investigation. The nanocomposites were characterized by AFM and DLS regarding their dimensions, while FT-IR and UV–VIS spectrometry were used for the optical properties and to reveal the binding of silver nanoparticles with chitosan. Their antibacterial effect was determined using a disk diffusion method on two bacteria strains, *E. coli* and *S. aureus*. The results indicate that, when using both methods, the nanocomposites obtained were below 100 nm, yet the antibacterial effect proved to be stronger for the nanocomposites obtained using sodium hydroxide. Furthermore, the antibacterial effect can be related to the nanocomposites’ sizes, since the smallest dimension nanocomposites exhibited the best bacterial growth inhibition on both bacteria strains we tested and for both types of silver nanocomposites.

## 1. Introduction

Antibacterial nanoengineered devices and tissue engineering showed comprehensive efforts to combine nanotechnology with materials sciences in order to restore, replace or improve the functions of damaged tissues and organs [1,2]. Infections induced by various microorganisms cause a large economic and medical problem, since the number of affected humans is increasing every year. The development of alternative methods based on nanotechnology for prevention or treatment has evolved lately due to the multiple advantages that biomaterials offer, alongside the classical strategies of treatment [3,4,5]. A faster and stronger effect, targeted administration and local administration are advantages offered using materials created in the laboratory for antibacterial biomaterials. Currently, noble metals such as silver, gold or zinc used for nanoparticles (NPs) or nanostructures synthesis [6] are considered potential agents to restrain microorganisms’ proliferation at damaged tissues [7,8,9]. Among them, silver nanoparticles (AgNPs) are considered a strong antibacterial agent studied lately due to their small sizes compared to major biological molecules, a property which allows them to perform proper diffusion through cell membranes of bacteria [10,11]. Their nanometric size offer them a larger surface area to volume ratio, which facilitates the interaction with bacteria cells [12]; therefore, size is an essential factor for this type of application. Furthermore, they possess interesting properties such as good optical activity, high surface energy [13] and easy manipulation regarding their syntheses. AgNPs are versatile regarding their antibacterial activity, meaning that they have a strong antifungal and antibacterial effect on different strains of bacteria. Studies reported that they present a strong antibacterial effect on Gram-negative bacteria such as *Escherichia coli* (*E. coli*) [14], *Vibrio anguillarum* [15], on Gram-positive bacteria such as *Bacillus cereus* [16], *Staphylococcus aureus* (*S. aureus*) [17] and even on fungi [18,19]. Since AgNPs are metal nanoparticles, the current trend is to combine them with more natural, biodegradable compounds to improve their biocompatibility, to enhance their chances to be tolerated better by tissues and to offer them support. For instance, chitosan (CH), a biodegradable polymer, is an excellent candidate for this type of nanocomposites, since it can improve the final properties of the material. Nanocomposites are preferable due to their multitude of benefits: they can combine the physical and antibacterial effect of AgNPs with the biological properties of natural polymers leading to a hybrid with superior properties [20]. 

Chitosan is a linear polysaccharide which has randomly β-(1-4)-linked D-glucosamine and N-acetyl-D-glucosamine groups in its structure [21,22]. It is a natural polymer obtained through the chitin deacetylation, an important component in crustaceans and shells [23,24]. The purity of chitosan is determined through the process of deacetylation; therefore, the extraction conditions are really important. Chitosan is an adaptable cationic polymer with a large amount of reactive amino and hydroxyl groups in its chemical structure. This property makes it a reactive reagent suitable for many types of applications as a biomaterial, including its integration in nanocomposites. Materials containing chitosan are non-toxic, biocompatible, renewable, biodegradable and easy to model because they are based on maneuverable polymers [25,26,27]. Also, they have a high absorption rate; therefore, films, sponges, and hydrogels can be manufactured using chemical or green chemical syntheses. Chitosan found applications in many domains such as in treatments for obesity [21], for dentistry [28], in drug delivery for improved encapsulation [29], in soft tissue engineering as gels for regeneration of skin or epithelium reconstruction [30,31] and in antibacterial applications together with AgNPs [32,33]. 

In AgNPs-CH nanocomposites, the reactive structure of chitosan is involved in interactions with metal ions, more precisely, with silver nanoparticles. The amino groups of chitosan interact with nanoparticles surfaces acting, at the same time, as a matrix and as a stabilizer of the newly formed network [34,35]. The bonds obtained in nanocomposites are based on the implementation of metal ions in chitosan chain through these amino groups. The proper interaction obtained between silver and a chitosan chain can be explained by chemical modifications appearing through the nitrogen atoms, which are rich in electrons [20]. Therefore, by understanding how these two form a strong chemical network, silver nanoparticles can be used to synthesize a new nanocomposite with high antibacterial efficiency in solutions with high viscosity like gels. This nanocomposite will present an enhanced surface charge and will be able to increase the interactions with bacteria membrane. Since we are taking about antibacterial behavior, regardless of the synthesis method chosen, the dimensions for the nanocomposite must remain in the nano domain because their inhibitory effect is bigger once their dimensions are smaller, as is confirmed in many studies in the literature, [36] being just one of them. The combination of properties obtained using chitosan and silver nanoparticles led to an increased attention of scientists since innovative synthesis methods can still be proposed. In a recent study, Mirda et al. (2021) [13] tested the antibacterial effect and the physical–chemical properties of a composite based on silver nanoparticles and chitosan obtained with a spherical shape. The synthesis involved the addition of NaOH in different volumes to obtain a clear idea about the antibacterial effect. The results of the study proved that once the NaOH concentration increases, the spheres have a more powerful effect on *S. aureus* and *E. coli* [13]. Another recent study performed by Bonilla et al. in 2022 [37] showed how AgNPs-CH nanocomposites synthesized with cysteine reduction act in induced chronic fungal infection in murine models. The in vitro results showed that *S. schenckii* and *S. brasiliensis* strains were inhibited by the materials fabricated, with a decrease in local inflammation. The release of AgNPs was improved with the chitosan integration and better tissue regeneration was achieved compared to simple AgNPs [37]. For instance, the implication of chitosan in increasing AgNPs efficiency was proved also by Shehabeldine et al. (2022) [38] in another study, where AgNPs-CH nanocomposites showed a higher inhibition zone compared to simple nanoparticles for Gram-negative and Gram-positive bacteria, while their cytotoxic effect remained the lowest [38]. Therefore, the synthesis of nanocomposites based on metal nanoparticles and chitosan is an interesting approach which can lead to materials fabrication using a fast procedure, with cost reduction and with valuable properties in antibacterial applications.

Recently, we developed simple Ag NPs using two different reducing agents and we saw that, through using trisodium citrate hydrate (TSC) as the main reducing agent in the chemical synthesis, we obtained nanoparticles of about 60 nm [39]. The aim of this article is to obtain nanocomposites based on AgNPs and chitosan using two different syntheses and three different chitosan concentrations, yet similar for a better comparison, and to identify their dimensions and their antibacterial effect, since these two properties are related. 

## 2. Materials and Methods

### 2.1. Materials

For this work, the materials were purchased from Sigma-Aldrich, Germany and VWR International, America in case of acetic acid, according to Table 1. The materials were used without any further purification. Three concentrations of chitosan were chosen to be used for two different nanocomposites syntheses: 0.3% *w*/*v*, 0.6% *w*/*v* and 0.9% *w*/*v*. Each of all three chitosan concentrations were dissolved in 1% acetic acid. Once the concentration of chitosan is higher, the color becomes more yellow.

**Figure 1:**
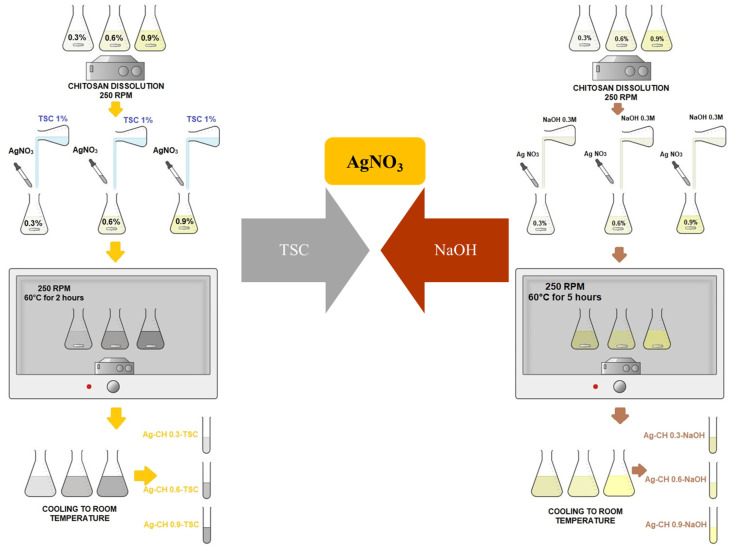
Chemical reductions in AgNPs-CH composites using two different agents.

The main goal of this study is to compare two synthesis protocols regarding the dimensions of nanocomposites based on silver nanoparticles in a chitosan hydrogel matrix using various conditions, various chitosan concentration and various reducing agents, yet comparable. Therefore, two syntheses using chemical reduction were proposed, as is described further in Figure 1 and Table 2, respectively.

### 2.2. Methods: Synthesis of AgNPs-CH Nanocomposites

For this paper, we performed six samples using two syntheses for the nanocomposites, as is described in the previous Figure 1, based on chemical reduction. For each sample, the volume chosen was 100 mL of chitosan solution. Also, there were prepared controls for chitosan solutions. All chitosan solutions were put on a BioSan shaker at 250 rpm at 50 °C for three hours in the oven. All chitosan solutions were taken out of the oven and were stirred in the shaker at 300 rpm at room temperature, overnight. The next morning, all the samples were completely dissolved; therefore, the next step was based on silver solution preparation. 10 mM AgNO_3_ solution was prepared with magnetic stirring for 15 min at room temperature.

In parallel, a 1% TSC solution and a fresh 0.3 M NaOH solution were prepared using ultrapure water as solvent. The six chitosan solutions for the nanocomposites were separated in the laboratory: three for the reduction based on TSC; three for the reduction using NaOH. The 10 mM AgNO_3_ was added drop by drop into each chitosan sample, while they were being stirred at 250 rpm. Immediately after, 10 mL TSC 1% was added drop by drop to the first three 100 mL solutions of AgNPs-chitosan so that the chemical reduction of silver ions can be achieved, while 1.5 mL of 0.3 M NaOH solution was added very slowly in the other three samples so that the chemical reduction of silver ions can also be achieved. The three samples with TSC were put in the shaker at 250 rpm and 60 °C for 2 h. The three samples using NaOH were put in the shaker at 250 rpm and 60 °C for 5 h. In case of NaOH, the AgNPs-CH solutions turned yellow in color, comparing to TSC, where the samples were grayer. All six solutions were let to cool down at room temperature and were kept in a dark spot for further analysis in 50 mL Falcon tubes.

### 2.3. Characterization Techniques

A DLS setup was performed for particle size determinations of nanocomposites. Beside it, AFM microscopy was used for dimensional properties with confirmation using DLS measurements. Furthermore, the viscosity of solutions was tested using an Ubbelohde viscosimeter for accurate results in DLS. ATR-FTIR spectroscopy was performed to show the integration of AgNPs in chitosan by identifying the specific chemical modifications that appeared in the nanocomposite. UV–VIS spectrophotometry was performed to analyze the stability of the solutions obtained and to confirm the AgNPs fabrication. The antibacterial test was made on two different types of bacteria, a Gram-negative strain, *E. coli* and a Gram-positive strain, *S. aureus* using a disk diffusion method.

#### 2.3.1. AFM Procedure

AFM offers various modes, each with distinct advantages. Contact mode provides high-resolution topographic information, but may result in sample scars and expedited tip wear [40]. Tapping mode enhances sensitivity to properties like stiffness and adhesion [41], while AFM can measure additional properties such as surface potential, magnetic fields and thermal conductivity [42].

Our study was conducted using an AGILENT 5500 AFM with a fixed sample on a substrate and a moving scanner. A small drop of each AgNPs-CH nanocomposite suspension was deposited on a quartz microscope coverslip, stretched thin and dried at 50 °C for 30 min. Tapping mode (or half-contact mode) at 512 × 512 pixels resolution was selected for scanning the samples. The AFM setup was isolated from vibrations and measurements were conducted during quieter evenings. Gwyddion software v2.29 on a Linux Mint platform processed AFM topography images, including background subtraction, scar correction, leveling and noise filtration. AgNPs-CH located in regions with large surface variations were excluded from size assessment.

We avoided automatic grain size measurements, so details and reasoning on this decision are presented in detail in our previous report [39]. We assumed that the vertical diameter is much closer to the actual diameter of the nanosized object. Consequently, this diameter, determined through the amplitude (topography) saved file, was employed for each nanoparticle. This methodology, consistent with assessments of nanosized object diameter in previous studies [43,44,45], demonstrated high consistency with diameters determined using an optical technique, specifically DLS. Therefore, it was adopted in the current study.

#### 2.3.2. DLS Data Processing Procedure

The DLS technique involves directing a coherent light beam onto particles in a liquid solvent, creating a coherent, interferential scattered light pattern [46,47,48,49]. A detector captures interference intensity, and the Data Acquisition System (DAS) records it as a time series [46,47]. Data processing aims to straightforwardly determine the average diameter of suspended particles [46,47,50], providing size information for nanoparticles and microparticles.

**Figure 2:**
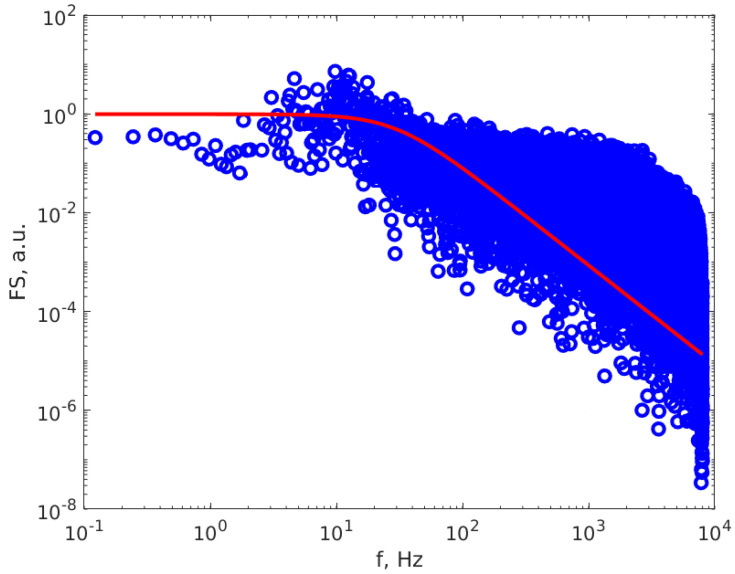
The PS calculated of a DLS time series (dots) and the Lorentzian line that best described the PS (continuous red line).

The experimental setup is presented in detail in one figure from our previous work (Figure 3 of [39]), together with the data processing procedure, based on fitting the scattered light intensity power spectrum (PS) using the Lorentzian line S(f), characterized by parameters a0 and a1, determined through least squares minimization [44,50]. Different from the setup and conditions used in [39], the scattering angle θ value is 20.14° and the DAS frequency is 16 kHz. The procedure described in [39] used the approximation of mono-sized particle distribution. The samples and the measuring conditions were different from those reported in [39]; therefore, we present below Figure 2, which illustrates the PS calculated using the FFT algorithm on a DLS time series (TS). Figure 2 reveals that the PS is well described by the Lorentzian line, within the inherent experimental errors caused by the finite DAS frequency and DLS time series lengths, thus confirming that the particle distribution is very close to mono sized.

Since DLS is a custom setup, the errors analysis is necessary to be done. Therefore, the error calculation for DLS diameters is described in Appendix A. We found that the relative error in assessing the diameters of particles was 13%. This error is relatively big but is consistent with the approach of simplifying the experimental setup and data processing procedure for DLS.

#### 2.3.3. Viscosity Measurements

The dynamic viscosity coefficient is one of the variables that must be known to assess the average diameter of the particles in suspension; therefore, it must be measured for solutions where the solvent is not water, for which the viscosity variation with temperature is well known. A rotating cylinder to measure viscosity is typically used to measure the viscosity of a fluid, but it requires a greater amount of fluid to fill the gap between the cylinder and the vessel. A capillary viscosity meter would be more appropriate if the amount of available fluid is smaller. 

An Ubbelohde type of viscosity meter was used for the measurements reported in this work. The work principle is based on Poiseuille’s equation, written as Equation (1) in this paper, which states that the volume flow rate of the fluid *Q* is proportional to the difference of pressure between the end of the tube Δ*p*, inversely proportional to the coefficient of dynamic viscosity of the fluid *η* and directly proportional to the 4th power of the radius of the capillary tube [51]. 

In Equation (1), *l* is the length of the capillary part of the instrument:

(1) $$Q=\frac{\pi ∆pR^{4}}{8\eta l}$$

By assuring that the same volume of different fluids flows through the same capillary under the same difference of pressure, we find that the dynamic viscosity coefficient of the fluid flowing through the capillary is directly proportional to the time *t* required for flowing [52,53,54], as illustrated in Equation (2).

(2) η=Const·t

If we differentiate Equation (2), we find that the relative error in assessing the dynamic viscosity coefficient is equal with the relative error in assessing the time of flow for the fluid volume.

(3) $$\frac{∆\eta }{\eta }=\frac{∆t}{t}$$

Time was not measured directly, but the experiment was recorded together with a digital chronometer displayed on a laptop screen. The recording was analyzed later and the moments of the beginning and of the end of the volume flowing through the capillary tube were assessed with a precision of 0.2 s and the time of flowing was computed. Water had been used as a reference fluid and therefore, the relative error for measuring the viscosity of the fluid was 1.8%. This is the value that was used in estimating the relative error for measuring the average diameter of the nanoparticles, as presented in Appendix A.

#### 2.3.4. UV–VIS Spectroscopy

For the investigation of all nanocomposites synthesized in this work, a Specord 210 Plus UV–VIS spectrophotometer provided by Analytik Jena was employed. The suspensions of AgNPs were introduced into a quartz cuvette, along with a water sample serving as a reference. We used UV–VIS spectroscopy to identify the Localized Surface Plasmon Resonance (LSPR), which is an indication of NPs presence in the sample [55].

#### 2.3.5. ATR-FTIR Spectroscopy

The Fourier Transform Infrared (FT-IR) spectrometry with ATR crystal is an important tool currently used in the structural analysis of nanocomposites. By examining the functional groups present in the suspension and the modifications that appear in the chemical groups comparing to the controls, valuable insights can be gained. In this study, an ALPHA spectrophotometer, manufactured by Bruker, was used. Equipped with an ATR crystal, the analysis was carried out within the wavelength range of 400–4000 cm−^1^, with 32 scan times at a resolution of 2 cm−^1^ for the all six AgNPs-CH suspensions and the absorbance of the samples was measured. These spectra offer valuable insight into the interactions taking place during the synthesis process between AgNPs and chitosan and illustrate the bonding of functional groups on the surface of AgNPs, as well. The use of FT-IR as a method for characterizing AgNPs-CH nanocomposites suspensions is supported by findings from other research studies, with refs. [35,37] being just some of them. As the AgNPs-CH nanocomposites are gels and water exhibits absorption over the whole wave number range, a relatively unconventional procedure was used. A drop of each aqueous suspension of the nanocomposites and of three concentrations of chitosan used in the reactions was deposited on a Petri dish and placed in a controlled temperature environment for 60 min to have the water evaporated. After this preparation step, the samples were removed and placed on the ATR crystal of the Bruker FT-IR spectrometer and an absorption spectrum was recorded for each of them.

#### 2.3.6. Antibacterial Test

The susceptibility of microorganisms to all six nanocomposites, AgNPs-TSC-CH and AgNPs-NaOH-CH, was tested using the disc diffusion method. For this research, two bacteria strains were tested, a Gram-negative (*E. coli*) and a Gram-positive (*S. aureus*) strain. Using two different types of bacteria for the antibacterial test means the effect is studied more precisely. The two microorganisms used are Selectrol reference strains from TCS Biosciences. Both bacteria were maintained in vials at −80 °C. The bacteria were seeded in Mueller Hinton II Agar 90 mm plates from Liofilchem. The disc diffusion analysis was conducted using sterile paper discs purchased from Merck company, in which 20 μL of each sample were pipetted for each bacteria strain. The loaded discs were allowed to dry for 10 min before being placed on the agar surface. The plates with media and bacteria seeded on them were maintained at 37 °C for 24 h. The antibacterial test was performed for both controls and nanocomposites samples, since we wanted to show that the antibacterial effect is accomplished only due to AgNPs and that the solvents used do not contribute to the antibacterial effect. Once the test is completed, the inhibition zone that appeared around the loaded disc is measured and based on the diameter of the inhibition zone, the sample that has the most powerful effect on the bacteria tested is established. The higher the inhibition zone, the higher the inhibitory effect of AgNPs.

In case of controls, 20 μL of TSC 1%, NaOH 0.1 M and CH 0.3% solutions for *E. coli* plate and the same amount for *S. aureus* plate were loaded because these are the reagents used in both syntheses. The CH 0.3% concentration was chosen because the smallest dimensions of AgNPs in the nanocomposites were obtained in this concentration. TSC and NaOH were tested in the same concentrations, as they were used in the synthesis, as well. After that, all three concentrations of AgNPs-TSC-CH and all three of AgNPs-NaOH-CH were tested for the *E. coli* strain and for the *S. aureus* strain.

## 3. Results and Discussion

### 3.1. AFM Results

**Figure 3:**
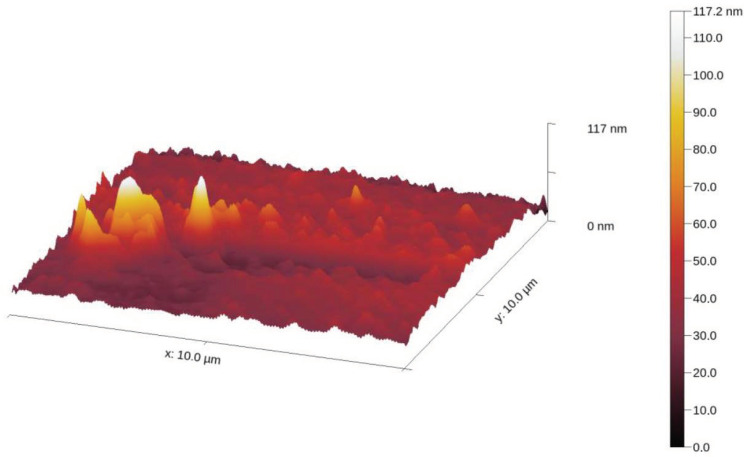
The topography of a region of the sample, illustrating a group of AgNPs-CH.

The samples were deposited as described above. Several scans were performed over different regions of the sample prepared in contact tapping mode with a soft cantilever. A sample from each AgNPs-CH was prepared in the same way, but drying was accelerated by placing it on a warm substrate at 50 °C, resulting in a solid AgNPs-CH. A 3D topography of a scanned region of AgNP-TSC-CH0.9 deposited on the quartz coverslip is presented in Figure 3.

We notice a group of AgNPs-CH in the left part of the image together with the chitosan grains spread on the rest of the coverslip. We also notice that the height of the nanocomposites is between 90 and 100 nm, according to the height color bar on the right part of the image.

**Figure 4:**
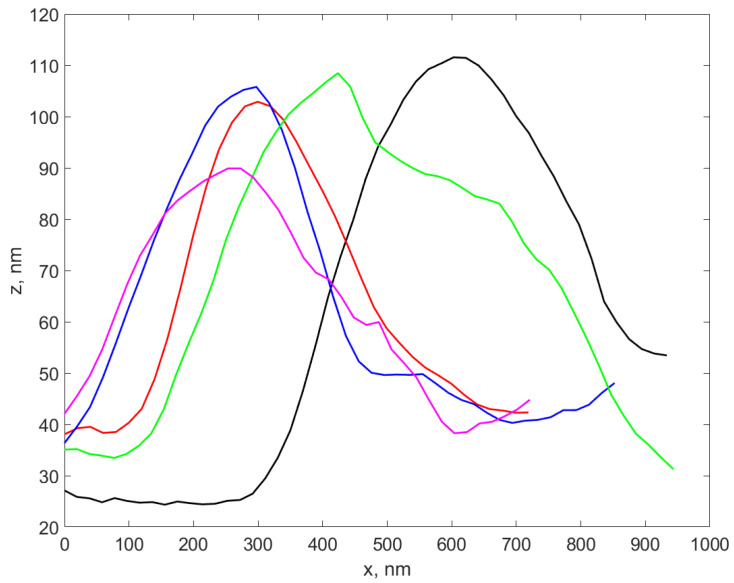
The plot of four profiles extracted over different AgNP-TSC-CH0.9 from the scanned sample, located in different regions of the sample.

Figure 4 illustrates several profiles extracted over different AgNP-TSC-CH0.9 regions of the sample, as described in Section 2.3.1.

**Figure 5:**
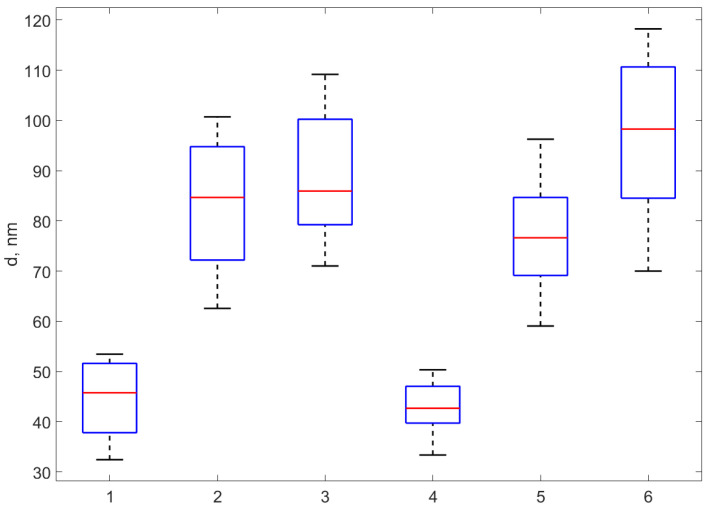
The boxplot of the diameters assessed as the height of the profile peaks extracted over 37 NPs from over each of the six samples. The boxplots correspond, from left to right, to AgNP-TSC-CH0.3, AgNP-TSC-CH0.6, AgNP-TSC-CH0.9, AgNP-NaOH-CH0.3, AgNP-NaOH-CH0.6, AgNP-NaOH-CH0.9.

We notice that the height of the profiles is different from each other, yet they are in the range of tens of nm. A total number of 37 profiles were carefully extracted from all six samples and the height of each profile was assessed as described in Section 2.3.1. The standard deviation (weighted at N-1) for each set of profiles was calculated and was considered the error in assessing the diameter using the AFM procedure, while the average of the height values in each set was considered to be the AFM diameter of the nanocomposite in that sample. The results are presented in the fourth column of Table 3. A boxplot of the AFM diameter values for the six sets corresponding to the six samples is presented in Figure 5.

### 3.2. DLS Particle Sizing Results

The DLS procedure previously described was used to process the TSs recorded on the aqueous suspension of all six nanocomposites, which are AgNP-TSC-CH0.3, AgNP-TSC-CH0.6, AgNP-TSC-CH0.9, AgNP-NaOH-CH0.3, AgNP-NaOH-CH0.6, AgNP-NaOH-CH0.9. Table 3 presents the average diameter assessed using the DLS procedure and the AFM procedure and the concentrations of nanocomposites.

Considering the AgNPs with a spherical form in a solution of volume V, a density (ρ_Ag_) of 10.49 g/cm^3^, a molar mass (μ_Ag_) of silver of 107.87 g/mol, N_A_ as Avogadro’ number and N_Ag_ as the number of initial Ag moles used in syntheses, the concentrations (C) of AgNPs-CH nanocomposites in the final solution involved in the syntheses were determined using the AFM particle size information (D) because the AFM diameter is closer to the real diameter [44]. We reached and used Equation (4) and the results are presented in Table 3.

(4) $$C=\frac{6{\times N}_{Ag}{\times \mu }_{Ag}}{\pi \times \rho_{Ag}{\times D}^{3}\times V{\times N}_{A}}(\frac{mol}{L})$$

We believe that it is worth emphasizing the distinction between DLS diameters and physical diameters assessed using the AFM technique. The DLS diameter signifies the size of a spherical particle diffusing at a rate equivalent to the particles generating the TS. It is noteworthy that nanoparticles of diverse shapes, including nanorods or irregular forms, diffuse in a fluid, resulting in a DLS TS and, consequently, a hydrodynamic diameter [56,57,58]. Moreover, the hydrodynamic diameter is slightly larger than the physical diameter for all particle types, attributed to the potential attraction of fluid molecules to nanoparticles via electrostatic forces between the negatively charged nanoparticle surface and the positive region of polar solvent molecules.

Additionally, the concept of a perfectly monodispersed size distribution is an idealized abstraction and not a realistic occurrence in the real world. The departure from this ideal represents a more-or-less accurate approximation of the actual particle size distribution. In the examination of Figure 2, it is evident that the fitted Lorentzian line (depicted as the red line) effectively characterizes the particle size data uniformly distributed around the line. The data displays a distinct plateau with a well-defined turnover point, affirming the suitability of the monodispersed approximation.

Furthermore, it is imperative to recognize that the average diameter is not a simple arithmetic mean of individual diameters, as the light scattering intensity follows the 6^th^ power of the diameter [59,60]. The interference landscape is primarily influenced by the largest particles in suspension. In situations where the particles are nearly monodispersed, the DLS diameter essentially signifies the average hydrodynamic diameter. Conversely, in polydisperse scenarios, the particle size, when depicted in a double logarithmic scale as in Figure 2, appears as a sum of Lorentzian lines rather than a single line and the DLS diameter reflects the average of the largest diameters. Nevertheless, in our investigation, the particle size is aptly represented by a single line and the DLS diameter is slightly smaller than the AFM diameter. The AFM diameter functions as validation for the size determined using the DLS. Considering the relatively limited number of profiles, obtaining another set across distinct nanoparticles may yield a slightly different average diameter and yet is comparable to the presented findings. The AFM diameter results are close to the DLS diameter measurement results; therefore, we are confident that the measurements were accurate, within the inherent error of each method.

Moreover, the DLS and AFM results synthetically presented in Table 3 indicate that the diameter of all nanocomposites is below 100 nm, making them suitable for biomedical applications. We also notice that the AgNPs-CH diameter increases with the concentration of chitosan for both reduction procedures, with TCS and with NaOH, and we interpret this feature as an indication that chitosan molecules are bound to the AgNPs surface. As chitosan concentration increases, the thickness of the surface layer increases as well, which is in good agreement with the results of the UV–VIS spectrometry and with the FT-IR spectroscopy results, presented in the next subsections.

### 3.3. UV–VIS Results

The UV–VIS absorption spectra of nanocomposites are influenced by their size, particularly affecting the position and intensity of their LSPR peak in the UV–VIS region. Upon excitation with incident light, the collective oscillation of conduction electrons in the metal induces LSPR, leading to significant absorption and light scattering at specific wavelengths. The LSPR peak characteristics, dependent on factors such as nanoparticle size and shape, exhibit a blue shift towards shorter wavelengths with a decrease in AgNPs’ size, as reported in various studies [61,62]. Quantum confinement effects, arising from electron confinement within a smaller volume, contribute to this observed blue shift. Studies, including refs. [61,62], consistently observe a systematic blue shift in the UV–VIS absorption spectra of AgNPs as their size decreases. This phenomenon underscores the intricate interplay between nanoparticle size, shape and composition in shaping absorption spectra. Additionally, factors like interparticle interactions, surface ligands, and dielectric environment further contribute to the nuanced features of AgNPs’ absorption spectra [61]. 

**Figure 6:**
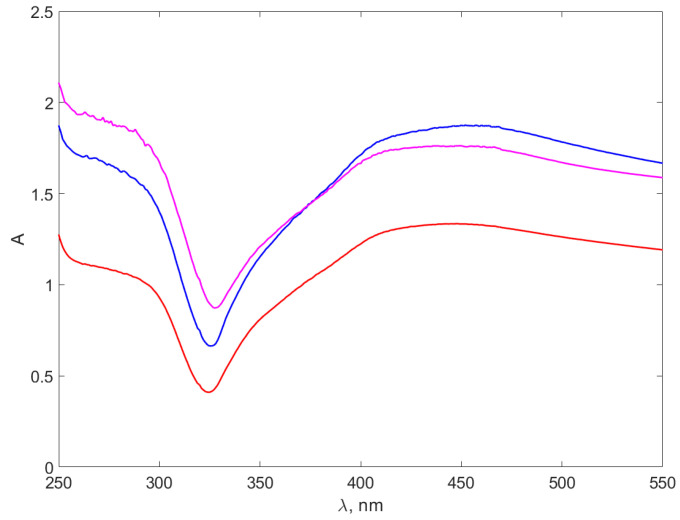
The absorption spectra of the AgNPs-TSC-CH. The red line stands for the AgNP-TSC-CH0.3 spectrum, the blue line for AgNP-TSC-CH0.6 and the purple line for AgNP-TSC-CH0.9.

**Figure 7:**
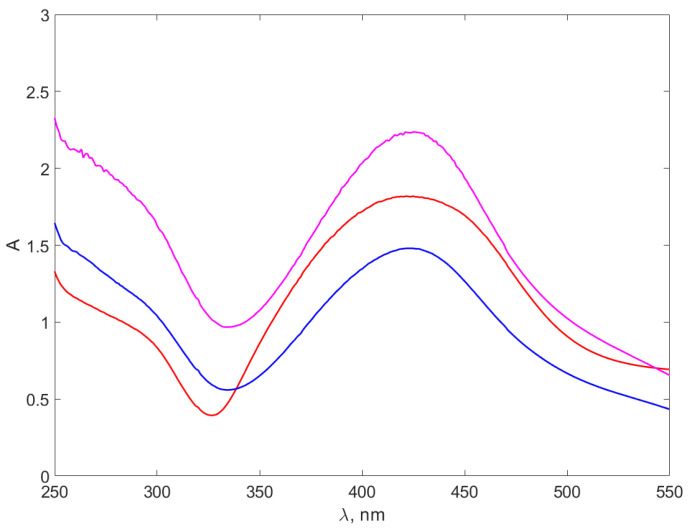
The absorption spectra of the AgNPs-NaOH-CH. The red line stands for the AgNP-NaOH-CH0.3 spectrum, the blue line for AgNP-NaOH-CH0.6 and the purple line for AgNP-NaOH-CH0.9.

We performed UV–VIS measurement for all pure chitosan samples; however, the results did not indicate absorption peaks and, therefore, we decided to present only the data collected for the nanocomposites. Figure 6 and Figure 7 illustrate the UV–VIS absorption spectra for the synthesized nanocomposites.

The range of the spectrum below 250 nm, demonstrating the pronounced absorption of UV electromagnetic radiation by water molecules, is omitted from Figure 6 and Figure 7.

Figure 6 of AgNPs-TSC-CH composites presents wider absorption peaks around 450 nm, aligning with the absorption peaks reported in the literature for AgNPs [61,62,63,64,65]. The peaks described in ref. [63], occurring at slightly smaller wavelengths, are consistent with the smaller size of the synthesized AgNPs. The position and the shape of the peaks are consistent with the reports of [39] on AgNPs UV–VIS spectra, as well.

The peaks in Figure 7 on AgNPs-NaOH-CH are slightly different from the peaks in Figure 6, as they are better defined and narrower. Moreover, they are located around 420 nm, with roughly 30 nm towards smaller wavelength. This appears surprising, considering that the dimensions of the DLS for nanocomposites presented in Table 3 appear to be very similar for the AgNPs-TSC-CH and AgNPs-NaOH-CH, differing with chitosan concentration. A possible explanation might be that the chitosan bond with the AgNPs is different for the two types of nanocomposites, consequently producing a shift of the absorption maximum of the LSRP and a difference in the shape of the peaks. It can be observed that peaks for Ag^+^ ions solutions are not present in the spectra and, therefore, the syntheses were complete.

The calculations presented in [61], employing various models, emphasize the impact of shape on the absorption peak position for 60 nm diameter volume oblate spheroids. The authors note that a larger axes ratio shifts the peak position towards smaller wavelengths.

The absorption spectra of the nanocomposites reveal LSRP, which is a strong confirmation of the AgNPs presence in the nanocomposites and confirms the tens of nm range measured with DLS and confirmed using the AFM measurements, as well. 

### 3.4. FT-IR Results

**Figure 8:**
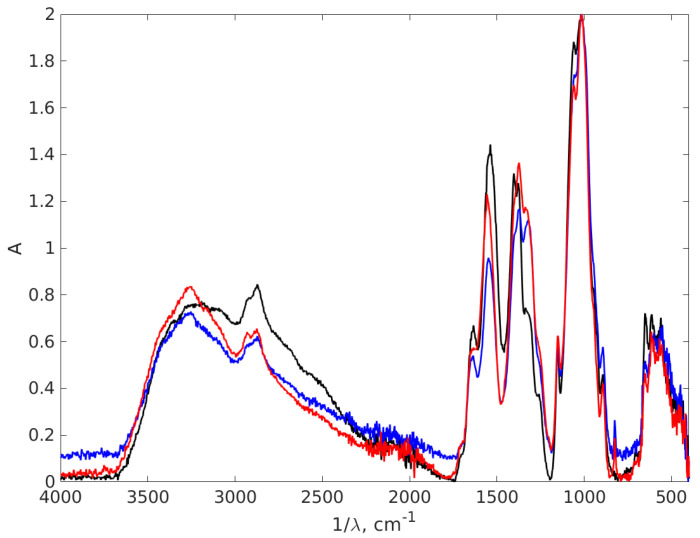
The FT-IR absorption spectra of the chitosan and AgNPs-CH with 0.9% chitosan concentration. The black line stands for chitosan 0.9%, the red line AgNP-TSC-CH0.9 and the blue line for AgNP-NaOH-CH0.9.

An FT-IR analysis was conducted to ascertain the various functional groups from the nanocomposites formed and to show the bindings between chitosan and AgNPs, as depicted in Figure 8.

**Figure 9:**
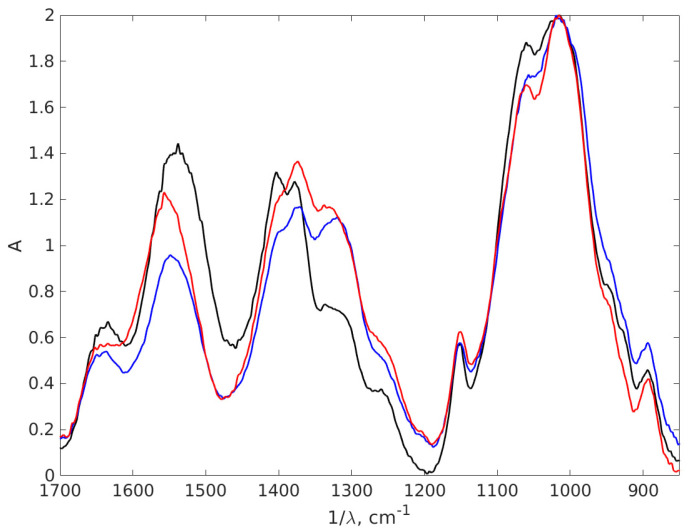
The zoomed FT-IR absorption spectra of the chitosan and AgNPs-CH with 0.9% chitosan concentration covering the wavelength interval 850–1700 cm^−1^. The black line stands for chitosan 0.9%, the red line AgNP-TSC-CH0.9 and the blue line for AgNP-NaOH-CH0.9.

The first indication about the formation of nanocomposites is based on the appearance of a large peak between 3500 and 3200 cm^−1^, with a maximum at 3252 cm^−1^, for AgNP-TSC-CH0.9 and AgNP-NaOH-CH0.9which it is not registered for the chitosan sample. This peak is associated with vibrational O-H groups and N-H stretching [66,67,68,69], since AgNPs are trapped in a chitosan matrix through exactly these types of bonds: the N atom from NH_2_ and O atom from OH group. This result suggests the stabilization of the composites. This sharp peak is consistent with both AgNPs plots; therefore, it is suggested that there is formation of AgNPs in the chitosan matrix using both methods. The bond at 2880 cm^−1^ represents symmetric C-H stretching [68], recorded due to the organic phase present in all three samples associated with the chitosan matrix. The large region of peaks recorded between 3000 cm^−1^ and 2800 cm^−1^ is associated with CH_2_-, CH_3_- vibrational groups, specific chemical groups for the polysaccharide structure [70]. The shifts that were recorded strongly suggested the formation of a nanocomposite. A zoomed FT-IR spectra is illustrated in Figure 9 to better depict the area. 

Both AgNPs-CH nanocomposites show a shift of the chitosan peak from 1544 cm^−1^ to 1636 cm^−1^ due to interactions formed between chitosan and AgNPs. These shifted peaks are associated with bending N-H vibrations from -NH_2_ amine groups [68,71,72,73], a result which suggests the interaction between chitosan and AgNPs. This stretching may appear due to the electrostatic interaction of Ag with the -NH_2_ group of chitosan [70]. Another shift between chitosan and nanocomposites appears around 1400 cm^−1^. The binding found in spectrum from 1402 cm^−1^ for chitosan is shifted to 1374 cm^−1^ for AgNP-TSC-CH0.9 and AgNP-NaOH-CH0.9. These bindings correspond to the C-N stretching appearing most probably due to the same interactions. A modification that appears around this vibration confirms that nanoparticles are trapped in chitosan matrix. Furthermore, the double peak observed here around 1400 cm^−1^ in case of chitosan is reduced for both nanocomposites to only one peak, as it can be observed in the zoomed spectrum. This is also an indicator that strong interactions between AgNPs and the chitosan rings are obtained in both nanocomposites fabricated. 

**Figure 10:**
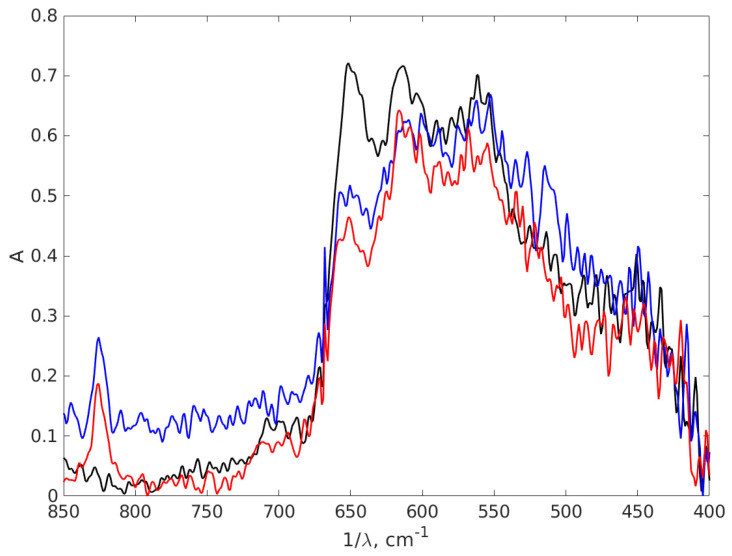
The FT-IR absorption spectra of the chitosan and AgNPs-CH with 0.9 chitosan concentration covering the wavelength interval 400–850 cm^−1^. The black line stands for chitosan 0.9%, the red line AgNP-TSC-CH0.9 and the blue line for AgNP-NaOH-CH0.9.

In FT-IR spectroscopy, the fingerprint region of the infrared spectrum refers to the lower wavenumber (higher frequency) portion of the spectrum, typically ranging from about 1500 to 500 cm−^1^. This contains unique and complex absorption bands that are highly specific to the molecular structure of a substance. Figure 10 presents another part of the IR absorption spectra of the chitosan and AgNPs with 0.9% chitosan concentration, covering the wavelength interval 400–850 cm^−1^, which is a part of the fingerprint region of the spectrum, to highlight the difference between the spectra in this part, which are dense in peaks that often overlap. The fingerprint region is less susceptible to interference from functional groups and solvents, making it useful in the analysis of complex mixtures.

Examining Figure 10 we notice in the left part of the spectrum two peaks at 825 cm^−1^ for both AgNP-TSC-CH0.9 and AgNP-NaOH-CH0.9, which are not present in the chitosan spectrum. Another peak can be found in chitosan at 651 cm^−1^ with a smaller intensity in the spectra of AgNP-TSC-CH0.9 and of AgNP-NaOH-CH0.9. Moving to the right of the spectrum, in Figure 10, we notice that the peak in chitosan located at 562 cm^−1^ is shifted at 567 cm^−1^ for AgNP-TSC-CH0.9, which is a very small shift and yet, it is meaningful because the spectra were recorded with a resolution of 2 cm^−1^. In other studies, the peak at 615 cm^−1^ is associated with an Ag-Ag interaction [74], but we believe that this peak may be attributed to C-H bonds outside the plane, which are present in the molecules, in agreement with [75]. The shifts in the positions of the absorption peaks in the FT-IR spectrum and the new peaks for both nanocomposites as compared to the chitosan spectrum are a strong indication that the AgNPs are bound to the functional groups of the chitosan matrix and form together a nanocomposite.

### 3.5. Antibacterial Test

**Figure 11:**
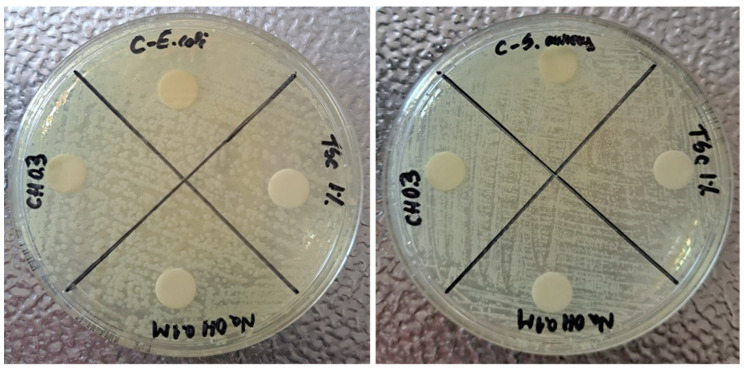
Antibacterial test for reagents used in reactions (controls): TSC 1%, NaOH 0.1 M, CH 0.3% using *E. coli* and *S. aureus* strains.

Figure 11 shows the results for the antibacterial test performed on controls for *E. coli* and *S. aureus*. The aim of testing these controls was to exclude their implication in the possible antibacterial effect of nanocomposites. The choice of CH 0.3% for these controls is based on the smallest nanoparticles’ dimensions obtained for this concentration. The idea is sustained by the affirmation that the antibacterial effect is stronger once the nanoparticles are smaller. The tests realized on controls proved that the reagents introduced in syntheses, TSC 1%, NaOH 0.1 M and CH 0.3%, do not have any antibacterial effect and do not inhibit any type of bacteria strain. It can be observed that the bacteria colonies are present all over the plate, which is also true in the case of chitosan. The C dial plates, which contain controls for the reactions, are based on empty sterile discs placed on the agar plates which proves that the tests are valid. Therefore, any possible bactericidal effect in future tests will only be due to the nanocomposites fabricated based on silver nanoparticles.

**Figure 12:**
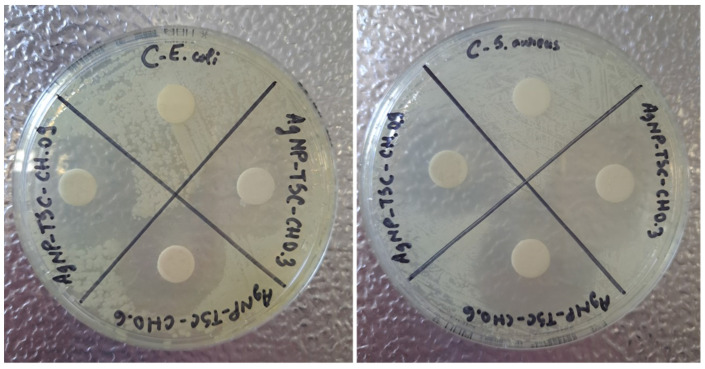
Antibacterial test using *E. coli* and *S. aureus* strain for AgNP-TSC-CH0.3, AgNP-TSC-CH0.6 and AgNP-TSC-CH0.9.

In the same condition, the diffusion disk method was employed for all six nanocomposites fabricated. The first analysis presented is for the susceptibility of both microorganisms to AgNPs-TSC-CH samples. The results are presented in Figure 12. 

For both bacteria strains, *E. coli* and *S. aureus*, the C dial of the plate validates the result, since it is a reaction control. The results obtained show that, in case of the control, the bacteria growth was not inhibited, while in the case of all three samples AgNPs-TSC-CH the microorganisms’ growth was stopped, proving their antibacterial effect. The inhibition zones for all chitosan concentrations are represented as a halo around the discs where bacteria were not allowed to proliferate and were killed. Therefore, the antibacterial effect is proved for these samples based on TSC and chitosan using their ability to inhibit bacteria proliferation. It is visually noticeable that there are some differences between the diameter zones depending on the chitosan concentrations. The inhibition zones’ diameters are presented in Table 4.

**Figure 13:**
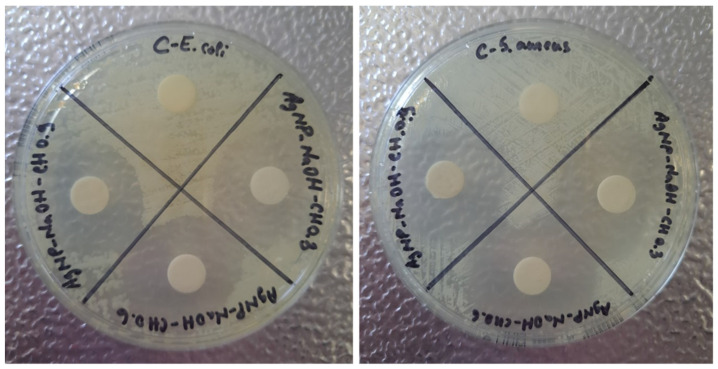
Antibacterial test using *E. coli* and *S. aureus* strain for AgNP-NaOH-CH0.3, AgNP-NaOH-CH0.6 and AgNP-NaOH-CH0.9.

Figure 13 presents the susceptibility of microorganisms to AgNPs-NaOH-CH samples. The results obtained after the incubation show that the test is valid, since in the case of reaction control, bacteria growth was not inhibited. This result, as in the case of previous samples, is an expected one, since the control contains an empty disc. For both *E. coli* and *S. aureus* strains, the inhibitory effect of samples based on NaOH and chitosan in all 0.3%, 0.6%, 0.9% concentrations is clearly observed in the plates. The halos around the loaded discs possess a large diameter, which suggests a strong antibacterial effect performed by these nanocomposites.

The increase of chitosan concentration in nanocomposites has a low impact on the antibacterial effect, as it is visible right from the dials. This difference is confirmed using the diameters of the inhibition zones presented in Table 4 and yet, it is observable also from the plate. In this case and in the case of AgNPs-TSC-CH as well, there are some small differences between the samples. These results suggest that the inhibitory effect on *E. coli* and *S. aureus* is due to the silver nanoparticles formed. This affirmation is also sustained by the results obtained in control tests, where chitosan proved to have no individual effect on bacteria strains. Yet, for the samples synthesized with NaOH, the antibacterial effect is stronger. Another aspect observed in this antibacterial test using diffusion in agar plates is that in case of the lowest concentration of chitosan, the halo around the loaded disc is bigger, a fact confirmed with the data obtained in the diameters’ measurements from Table 3, where the smallest dimensions were obtained for nanocomposites with chitosan 0.3%. Both syntheses proposed for the nanocomposites showed strong antibacterial effect for *E. coli* and *S. aureus* strains.

Once the antibacterial effect on *E. coli* and *S. aureus* were clearly confirmed, the diameters of inhibition zones were measured using a ruler for each bacteria strain and each sample. A higher diameter inhibition zone suggests there is a greater antibacterial effect for the sample. Furthermore, the results are presented in Table 4.

The data collected show that the highest antibacterial effect is confirmed in the samples with the lowest chitosan concentration and the smallest dimensions, AgNP-TSC-CH0.3 and AgNP-NaOH-CH0.3, where the inhibition zones are the largest. There are no major differences between the samples for the same bacteria strain. A real difference is observed between the two bacteria strains. The antibacterial effect is higher for *S. aureus* compared to *E. coli*, since the inhibition zones are larger in diameter in the case of *S. aureus* for all samples.

The nanocomposites with the lowest chitosan concentration, which had the smallest dimensions in their DLS results, inhibit bacteria proliferation in the highest percentage. For TSC reducing agent, the inhibition of *E. coli* was confirmed based on a 31 mm diameter inhibition zone, while the inhibition of *S. aureus* was identified based on a 32 mm diameter halo in the disk diffusion test. The same observation was recorded for the samples in which NaOH was used as well, because the smallest particles showed a higher antibacterial effect. Furthermore, AgNP-NaOH-CH0.9, the sample with the lowest dimension, 46.6 nm, exhibits a stronger antibacterial effect, since the inhibition zones for both bacteria strains are greater compared to the other samples. The diameters obtained for both sets of samples are similar in DLS analysis (TSC versus NaOH); therefore, the results can be correlated with the antibacterial test where a decrease of the effect appears once the dimension increases. The antibacterial effect of our AgNPs composites is similar with reports in the literature on AgNPs’ antibacterial effect [76], on AgNPs composites [77], and on Ag_2_O NPs [78], to mention just some of them. The antibacterial effect was confirmed for all samples and yet, by comparing both syntheses method, we can state that the implication of NaOH in the reducing reaction increases the antibacterial effect of the samples.

## 4. Conclusions

This work presents two procedures for the manufacturing of nanocomposites based on AgNPs and chitosan. The synthesis method was a chemical one, using two different conditions, one based on TSC and the other one with NaOH. Regarding their characterization, the application of both AFM and DLS for particle sizing reveals that the AgNPs-CH nanocomposites synthesized using the two reduction procedures have average diameters from 40 to 100 nm, so they are suited for biomedical applications. Worth mentioning is that for both reduction methods the nanocomposite diameters increase once the chitosan concentration increases from 0.3% to 0.9%. We view this characteristic as an indication that the chitosan molecules are bond to the AgNPs and that nanoparticles are found in the chitosan matrix. The UV–VIS spectroscopy investigation revealed that AgNPs-TSC-CH presents wider absorption peaks around 450 nm, while the absorption peaks of AgNPs-NaOH-CH are better defined, narrower and located around 420 nm, with roughly 30 nm towards smaller wavelength. This feature is an indication that the chitosan binding to the AgNPs is different for the two types of nanocomposites. The FT-IR analysis substantiates these observations, revealing characteristic peaks associated with functional groups of chitosan, slightly shifted in the nanocomposites’ samples. The results of the antibacterial test revealed that both nanocomposites inhibited the bacteria strains tested, *E. coli* and *S. aureus* and yet, some differences were noticed. We found that once nanocomposites dimension decreases, their antibacterial effect increases. The sample with the smallest dimension of 46 nm, AgNP-NaOH-CH0.9, showed the strongest antibacterial effect on both bacteria tested, *E. coli* and *S. aureus*. Therefore, the materials we synthesized can be successfully used in antibacterial applications.

## Figures and Tables

**Table 1:** Materials used in this research for AgNPs-CH nanocomposites.

Reagent	Chemical Formula	Purity	Form
**Silver nitrate**	AgNO_3_	≥99.8% ACS	powder
**Trisodium citrate dihydrate (TSC)**	C_6_H_5_Na_3_O_7_·2H_2_O	99%	powder
**Sodium hydroxide (NaOH)**	NaOH	97%	powder
**Chitosan low molecular weight (CH)**	[C_6_H_11_NO_4_]_n_	>75% deacetylation	powder
**Acetic acid**	C_2_H_4_O_2_	99.8%	liquid

**Table 2:** Chemical compositions and sample descriptions for a total volume of 100 mL.

Number	Sample	Chitosan, *w*/*v* (%)	AgNO_3_ (mM)	TSC 1%
**1**	AgNP-TSC-CH0.3	0.3	10	1
**2**	AgNP-TSC-CH0.6	0.6	10	1
**3**	AgNP-TSC-CH0.9	0.9	10	1
**Number**	**Sample**	**Chitosan, *w*/*v* (%)**	**AgNO_3_ (mM)**	**NaOH (mM)**
**4**	AgNP-NaOH-CH0.3	0.3	10	0.1
**5**	AgNP-NaOH-CH0.6	0.6	10	0.1
**6**	AgNP-NaOH-CH0.9	0.9	10	0.1

**Table 3:** The DLS and AFM diameters of nanocomposites, the error in assessing them and the nanocomposites concentrations.

No.	Sample	d DLS, nm	d AFM, nm	Molar Conc. AgNPs-CH, mmol/L
**1**	AgNP-TSC-CH0.3	48.8 ± 6.3	44.5 ± 7.1	3.7 × 10^−6^
**2**	AgNP-TSC-CH0.6	85.7 ± 11.1	82.7 ± 12.1	5.7 × 10^−7^
**3**	AgNP-TSC-CH0.9	98.5 ± 12.8	89.1 ± 12.0	4.6 × 10^−6^
**4**	AgNP-NaOH-CH0.3	46.6 ± 6.1	42.6 ± 5.2	4.2 × 10^−6^
**5**	AgNP-NaOH-CH0.6	92.0 ± 12.0	76.7 ± 10.3	7.2 × 10^−7^
**6**	AgNP-NaOH-CH0.9	100.8 ± 13.1	97.2 ± 14.8	3.6 × 10^−7^

**Table 4:** Inhibition zones obtained in the antibacterial test.

No.	Sample	Inhibition Zone for *E. coli* Strain	Inhibition Zone for *S. aureus* Strain
**1**	AgNP-TSC-CH03	31 mm	32 mm
**2**	AgNP-TSC-CH06	30 mm	31 mm
**3**	AgNP-TSC-CH09	30 mm	27 mm
**4**	AgNP-NaOH-CH03	32 mm	33 mm
**5**	AgNP-NaOH-CH06	32 mm	33 mm
**6**	AgNP-NaOH-CH09	31 mm	29 mm

## Data Availability

Data can be available upon request.

## Appendix A. Error Calculation for DLS Diameters

Given the customized nature of the DLS setup, where all parameters, including the DAS sampling rate, are adjustable to fulfill specific requirements, we consider that providing a concise error analysis is necessary. To evaluate the relative error in determining the particle *R*, we can represent the logarithm of *R* as illustrated in Equation (A1):

(A1) $$R=\frac{16\pi k_{B}n^{2}}{3\eta a_{1}\lambda^{2}}T\sin^{2}\frac{\theta }{2}$$

By grouping all the constants together, the differential of that factor becomes zero. Assuming that the sources of errors were the thermodynamic temperature (*T*) and the measuring angle (θ), we can express the logarithm of d as given in Equation (A2):

(A2) $$\ln\left(R\right)=\ln\frac{16\pi k_{B}n^{2}}{3a_{1}\lambda^{2}}-ln\eta +lnT+2\ln\left(\sin\frac{\theta }{2}\right)$$

By differentiating Equation (A2) and considering dη, d*T* and dθ as the experimental errors associated with the measurements, assuming errors to accumulate in the most unfavorable manner, we obtain Equation (A3):

(A3) $$\varepsilon_{R}=\frac{\Delta R}{R}=\frac{\Delta \eta }{\eta }+\frac{\Delta T}{T}+\frac{1}{\tan\left(\frac{\theta }{2}\right)}\Delta \theta$$

The error was 0.5 K for temperature measurement, with T being 20 °C (293.15 K). The sampling rate was 16,000 Hz, the detector-tube distance *x* was 5 cm, the scattering angle θ was 20.14° and the tube diameter was 1 cm, which caused an error in assessing the scattering angle θ. To assess the last term of Equation (A3), we notice that:

(A4) $$x=\frac{y}{tan\left(\theta \right)}$$

And by differentiating and reverting, we find that the last term of Equation (A3) is:

(A5) $$\frac{\Delta \theta }{\tan\left(\frac{\theta }{2}\right)}=\frac{\Delta x}{y}\cdot \frac{{sin}^{2}\theta }{tan\frac{\theta }{2}}$$

Replacing Equation (A5) in Equation (A3) we find:

(A6) $$\varepsilon_{R}=\frac{\Delta R}{R}=\frac{\Delta \eta }{\eta }+\frac{\Delta T}{T}+\frac{\Delta x}{y}\cdot \frac{{sin}^{2}\theta }{tan\frac{\theta }{2}}$$

The relative error is 1.8%, as described above. Replacing them, we found that the relative error in assessing the radius, hence the diameter of the particles, was 13%. This error is relatively big but is consistent with the approach of simplifying the experimental setup and data processing procedure for DLS.
